# Supplementary material for: Detection and Level Evaluation of Antibodies Specific to Environmental Bacteriophage I11mO19 and Related Coliphages in Non-Immunized Human Sera
Source: Antibiotics (Basel). 2023 Mar 15;12(3):586. doi: 10.3390/antibiotics12030586 (PMC10044619; doi:10.3390/antibiotics12030586)
Supplement: Supplementary file 1 [file antibiotics-12-00586-s001.zip › antibiotics-2260565-supplementary.pdf]

Supplementary material

Detection and level evaluation of antibodies specific to environmental bacteriophage I11mO19 and related coliphages in non-immunized human sera

Ewa Brzozowska [1*], Tomasz Lipiński [2], Agnieszka Korzeniowska-Kowal[1], Karolina Filik[1], Andrzej Górski[3] and Andrzej Gamian[1]

1. Laboratory of Medical Microbiology, Laboratory of Medical Microbiology, Department of Immunology of Infectious Diseases, Hirszfeld Institute of Immunology and Experimental Therapy, Polish Academy of Sciences, Weigla 12, 53-114 Wrocław, Poland; ewa.brzozowska@hirszfeld.pl, agnieszka.korzeniowska-kowal@hirszfeld.pl, karolina.filik@hirszfeld.pl, andrzej.gamian@hirszfeld.pl
2. Bioengineering Research Group, Łukasiewicz Research Network-PORT Polish Center for Technology Development, 54-066 Wroclaw, Poland; tomasz.lipinski@port.org.pl
3. Bacteriophage Laboratory, Hirszfeld Institute of Immunology and Experimental Therapy, Polish Academy of Sciences, Weigla 12, 53-114 Wrocław, Poland; gorski@hirszfeld.pl

*Correspondence: ewa.brzozowska@hirszfeld.pl; phone: +48 71 3371172.

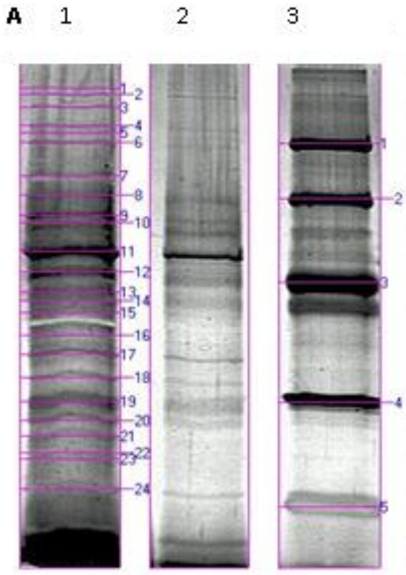

	MW-HF	
	L2	L4
1	121.000	94.000
2	118.065	67.000
3	111.609	43.000
4	102.804	30.000
5	99.283	20.000
6	94.587	
7	77.403	
8	68.531	
9	61.573	
10	59.286	
11	50.672	
12	45.401	
13	41.128	
14	39.680	
15	37.886	
16	35.207	
17	33.490	
18	31.802	
19	30.091	
20	28.548	
21	27.164	
22	25.568	
23	24.976	
24	21.899	

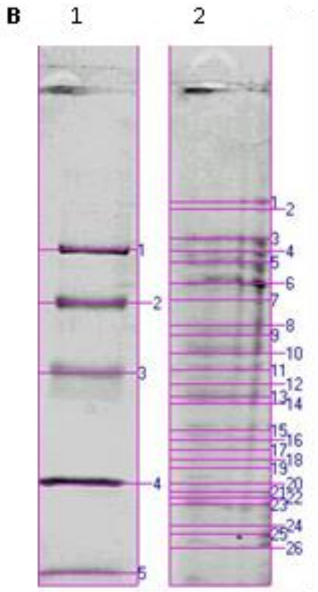

	L1	L2
1	94.000	118.158
2	67.000	114.605
3	43.000	99.684
4	30.000	93.251
5	20.000	87.276
6		76.389
7		68.278
8		58.368
9		54.758
10		48.571
11		43.711
12		40.465
13		38.140
14		37.058
15		33.992
16		33.161
17		32.422
18		31.734
19		31.150
20		30.000
21		29.286
22		28.773
23		28.094
24		25.839
25		24.864
26		23.170

Figure S1. Molecular weights of bacteriophage I11mO19 proteins (A) – lanes 1 and 2), 3) molecular weight standard. (B) Proteins of E. coli D- the host of I11m19 phage – lane 2, 1) molecular weight standard. Proteins were separated in a 12.5% gel by electrophoresis under denaturing conditions. Bacterial lysate proteins were stained with Coomassie Brilliant Blue R-250 and phage proteins with silver. Molecular weights were analyzed using Bio-Profile Bio-1D++ Windows Application V99.04.

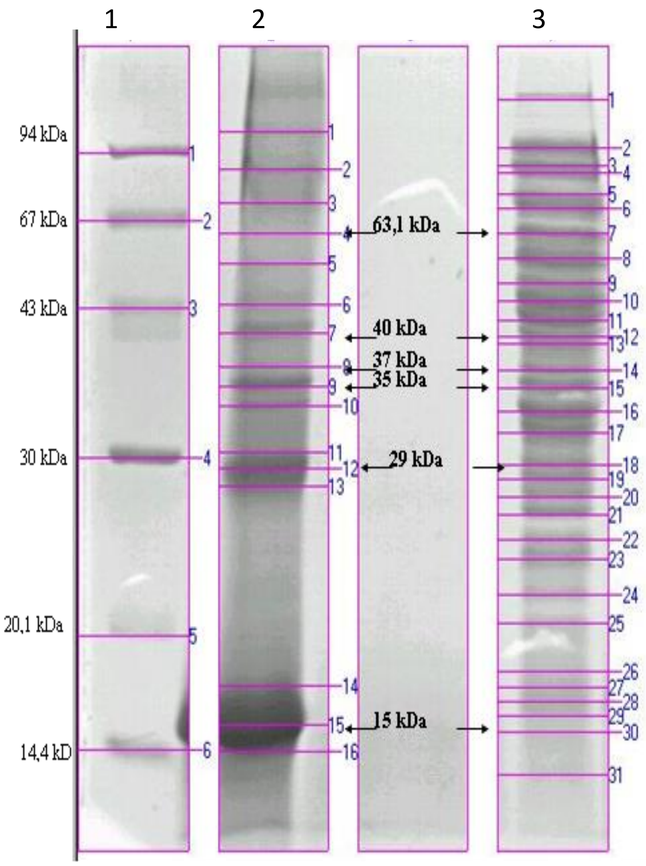

Figure S2. Comparison of ΦK1E bacteriophage (2) and E. coli K1 proteins (3). (1) Molecular weight standard. Proteins were separated in a 12.5% gel by electrophoresis under denaturing conditions. Proteins were stained with Coomassie Brilliant Blue R-250. Molecular weights were analyzed using Bio-Profile Bio-1D++ Windows Application V99.04

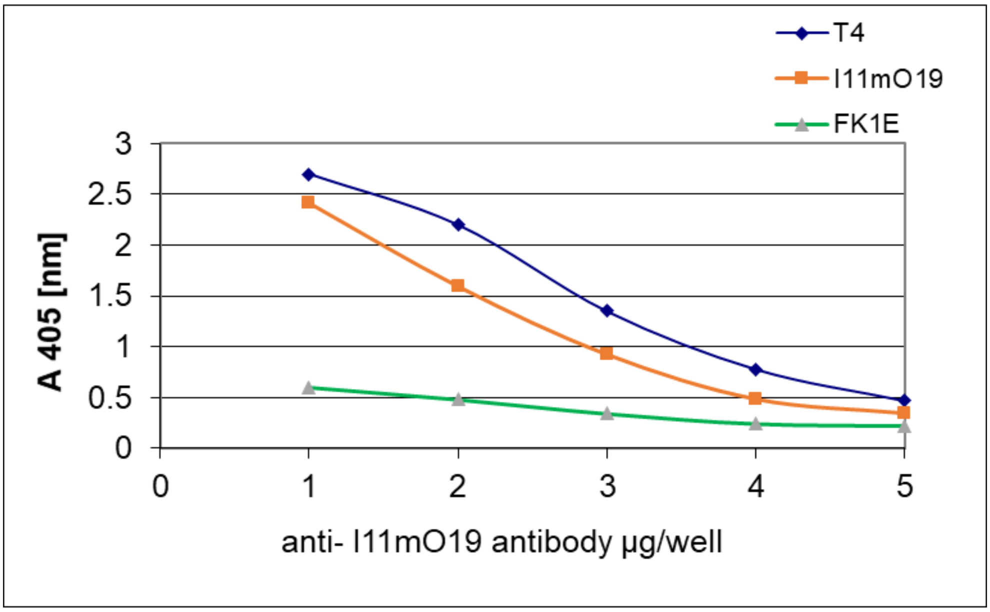

Figure S3. Immunoreactivity of isolated anti-I11mO19 antibodies with phage proteins; ϕK1E (green), I11mO19 (orange), and T4 (dark blue). The amount of phage proteins was 1.0 µg per well.

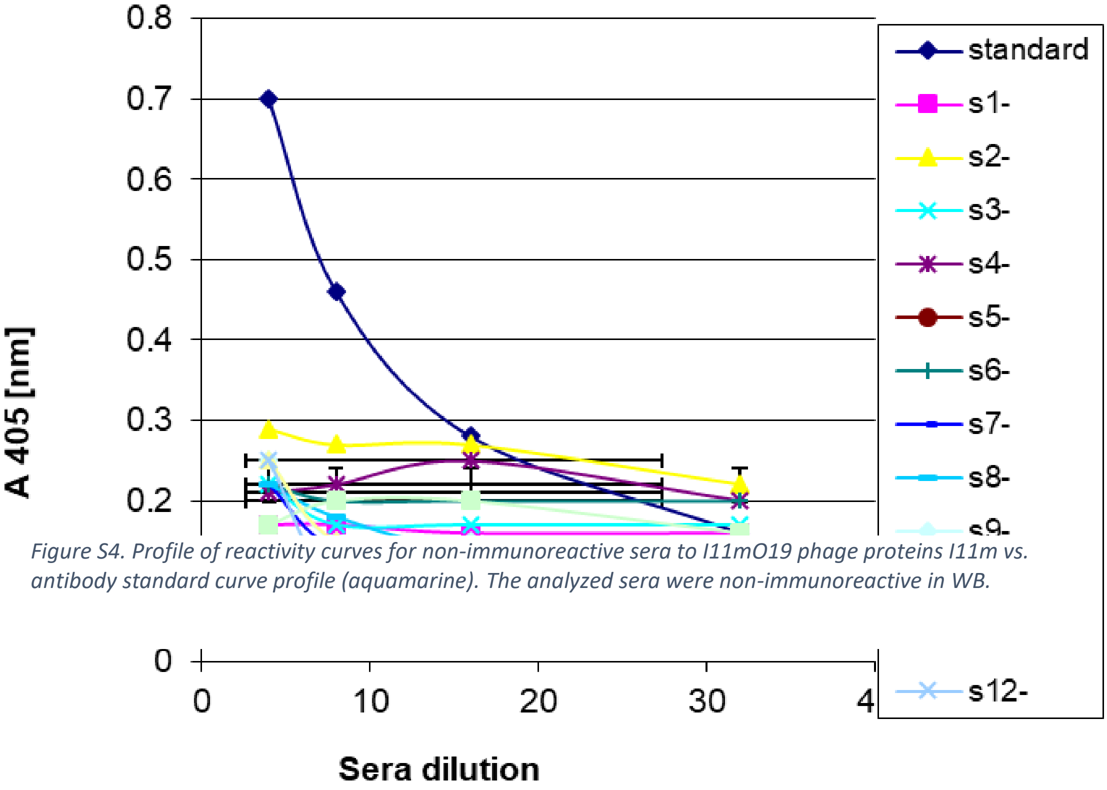

Figure S4. Profile of reactivity curves for non-immunoreactive sera to I11mO19 phage proteins I11m vs. antibody standard curve profile (aquamarine). The analyzed sera were non-immunoreactive in WB.

Figure S4. Evaluation of immunoreactivity of human sera determined as negative to I11mO19 phage in ELISA. Standard (dark blue) stands for anti-I11mO19 antibody isolated in affinity chromatography. 1.0 µg of I11mO19 phage proteins coated the plate well, and human sera were diluted 4; 8;16; and 32 times. The amount of 'standard' was in the range of 0.06 to 1.2 µg. The conjugate - goat anti-human IgG with alkaline phosphatase was diluted.1/10000.

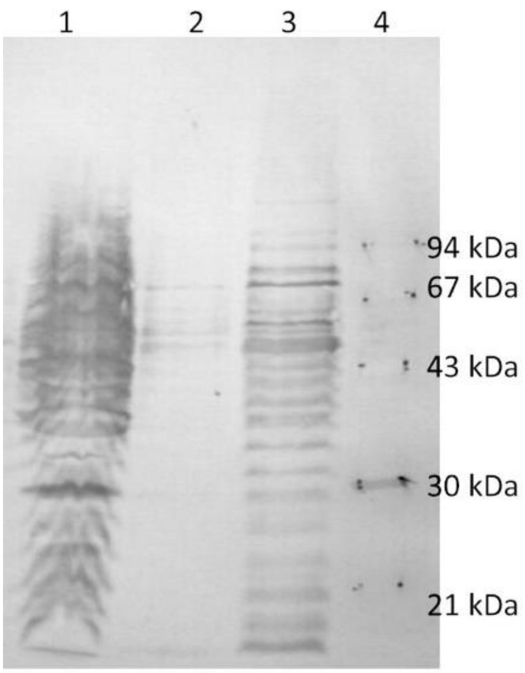

Figure S5. Immunoreactivity analysis of human serum to proteins of E. coli D – the host for I11mO19 phage (1) and phage I11mO19 (2) and the host's LPS (3). The human serum 250 times diluted.

Table S1. Analysis of molecular weights of bacteriophage T4 and E. coli B proteins separated by electrophoresis in a 12.5% polyacrylamide gel under denaturing conditions. Molecular weights were analyzed using Bio-Profil Bio-1D++ Windows Application V99.04. The same MW in phage and in bacteria protein's panel are marked in blue

**Molecular weights of bacteriophage T4 proteins [kDa]]**	**Molecular weights of *E. coli* B proteins [kDa]**
**133.7**	**96.8**
**123.8**	**90.7**
**115.3**	**74.0**
**101.1**	**57.9**
**79.6**	**51.1**
**74,0**	**45.4**
**68.2**	**40.5**
**56.2**	**35.6**
**46.3**	**32.9**
44.3	**31.6**
**38.3**	**27.2**
**35.6**	**23.3**
**33.9**	**19.8**
**30.9**	**16.5**
**22.5**	**12.6**
**14.8**	

Table S2. Analysis of molecular weights of bacteriophage F8 and P.aeruginosa proteins separated by electrophoresis in a 12.5% polyacrylamide gel under denaturing conditions. Molecular weights were analyzed using Bio-Profile Bio-1D++ Windows Application V99.04

**Molecular weights of bacteriophage F8 proteins [kDa]]**	**Molecular weights of outer membrane proteins *of Pseudomonas aeruginosa* [kDa]**
**92.83**	**96.54**
**57.64**	**85.48**
**47.98**	**75.81**
**35.7**	**72.38**
**27.82**	**65.35**
**15.86**	**58.07**
	**55.73**
	**45.28**
	**41.64**
	**40.28**
	**37.83**
	**32.12**
	**28.03**
	**27.15**
	**24.26**
	**21.54**
	**20.74**
	**19.11**
	**17.63**
	**16.35**
	**15.79**
	**15.05**

Table S3 Table showing the average A405 values obtained in the ELISA test in the reaction of the standard (i.e., anti-I11m antibodies)diluted from ¼ to 1/128, with I11m phage proteins.

**Standard dilution**	**Arithmetic mean of A_{405}**	**Standard deviation (sD)**
¼	0.765	0.086
$^1/_8$	0.527	0.053
$^1/_{16}$	0.315	0.026
$^1/_{32}$	0.157	0.029
$^1/_{64}$	0.082	0.010
$^1/_{128}$	0.034	0.004
